# Effect of dietary components on miRNA and colorectal carcinogenesis

**DOI:** 10.1186/s12935-018-0631-y

**Published:** 2018-09-06

**Authors:** Adewale Oluwaseun Fadaka, Babajide A. Ojo, Olusola Bolaji Adewale, Temitope Esho, Ashley Pretorius

**Affiliations:** 1 Department of Biotechnology, Faculty of Natural Sciences, University of the Western Cape, Cape Town, South Africa; 2 Department of Nutritional Science, Oklahoma State University, 301, Human Sciences, Stillwater, OK 74075 USA; 3 Department of Biochemistry, Afe Babalola University, P.M.B. 5454, Ado-Ekiti, Ekiti State Nigeria; 4 Institute of Biochemistry II, Medical Faculty, University of Cologne, Joseph-Stelzmann Str. 52, 50931 Cologne, Germany; 5 Biotechnology Innovation Division, Aminotek PTY LTD, Suite 2C, Oude Westhof Village Square Bellville, 7530 South Africa

**Keywords:** Colorectal cancer, microRNA, Biomarkers, Diet, Chemoprevention

## Abstract

**Background:**

Colorectal cancer (CRC) is one of the most common cancers diagnosed and among the commonest causes of cancer-related mortality globally. Despite the various available treatment options, millions of people still suffer from this illness and most of these treatment options have several limitations. Therefore, a less expensive, non-invasive or a treatment that requires the use of dietary products remains a focal point in this review.

**Main body:**

Aberrant microRNA expression has been revealed to have a functional role in the initiation and progression of CRC. These has shown significant promise in the diagnosis and prognosis of CRC, owing to their unique expression profile associated with cancer types and malignancies. Moreover, microRNA therapeutics show a great promise in preclinical studies, and these encourage further development of their clinical use in CRC patients. Additionally, emerging studies show the chemo-preventive potential of dietary components in microRNA modulation using several CRC models. This review examines the dietary interplay between microRNAs and CRC incidence. Improving the understanding of the interactions between microRNAs and dietary components in the carcinogenesis of CRC will assist the study of CRC progression and finally, in developing personalized approaches for cancer prevention and therapy.

**Conclusion:**

Although miRNA research is still at its infancy, it could serve as a promising predictive biomarkers and therapeutic targets for CRC. Given the ever-expanding number of miRNAs, understanding their functional aspects represents a promising option for further research.

## Background

Colorectal cancer is the third most commonly diagnosed cancer and the fourth leading cause of cancer-related deaths in the world [1]. It is the fourth most common cancer in South Africa in both male and female and the 6th most lethal of all known cancers [2]. Bray et al. [3] has predicted that there will be an increase in all CRC incidence cases from over 12 million in 2008 to about 22.2 million by 2030 [4]. About 24 million new cases of CRC was expected to be diagnosed by 2050, out of which 70% of these cases would be found in the developing countries [5]. Occurrence of this disease is greater in America and Europe when compared to low and middle-income countries. However, there is still high burden of this disease due to lack of early diagnosis of CRC as a result of limited resources in these low and medium-income countries, such as most African countries. Although, this is avoidable because CRC is one of the cancers that is almost 100% preventable [6], but most of the world’s population still lack information of this disease as well as its relationship with diet.

Treatment options for CRC is largely dependent on the stage of the tumor, that is, how far it has metastasized. A common non-invasive screening test currently employed is the Fecal Occult Blood Test (FOBT), but the test presents poor sensitivity and specificity [7]. Other screening tests such as the Fecal Immunochemical Test (FIT), the fecal DNA test and the plasma SEPT-9 gene methylation test [8], as well as colonoscopy [9] are in use. Some clinicians use the FOBT and colonoscopy together or at different times [10]. Serum biomarker test such as carcinoembryonic antigen (CEA), carbohydrate antigens (CA) 19-9, and CA 125 used for CRC diagnosis are also non-specific [11]. To date, colonoscopy is the most commonly used test in the detection of CRC, which has been found to reduce the risk of CRC by 30–75%, but the limitation to this technique is its high cost and invasiveness [12], which makes it ineffective in resource-limited settings. Chemotherapeutic agents that often used in post-surgery lack tissue selectivity. At early stage, CRC may not show obvious signs or symptoms such as colon and/or rectal bleeding, belly pain, change in bowel habit (diarrhea), constipation, stool narrowing, and sudden weight loss. This disease can be asymptomatic until latter stages when the cancer has metastasized [13]. Globally, the major challenge to CRC treatment is early detection, which makes the current treatment options to be administered so late, typically after the cancer has metastasized. If the cancer is detected early, and polyps are removed by surgery, this will reduce both the incidence and mortality cases of CRC. To achieve this, more non-invasive, selective and specific diagnostic tools which can detect the tumour at an early need to be reviewed.

Non-coding RNAs, most especially miRNAs, are attracting considerable interest, with increasing evidences on the role of miRNAs’ expression in CRC development and progression [14]. This has led to the use of miRNAs as therapeutic targets. Nevertheless, the mechanism through which a single miRNA controls gene networks by and the possible in vivo adverse effects of the miRNA and/or anti-miRNA are yet to be fully explored. As earlier mentioned, early CRC detection tools are faced with several challenges, thereby limiting the development of a standardized biochemical diagnostic approaches which are non-invasive, more sensitive and specific for CRC stages. Several factors have been linked to the disease risk factors but adopting a healthy lifestyle could be a preventable means. As a result of these, diet has been implicated in a crucial role in preventing CRC [15]. Therefore, diet-miRNA interplay and identification of the miRNAs that are expressed in CRC would be a focal point in this review.

## Colorectal cancer

**Fig. 1:**
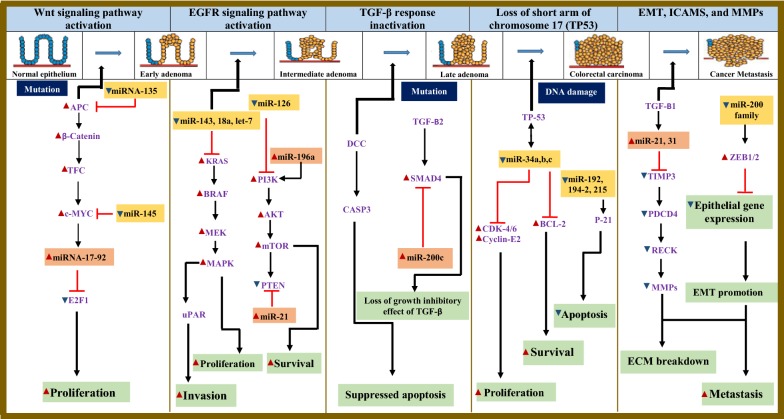
microRNAs and genetic/epigenetic alterations involved in adenoma-carcinoma sequence. Red arrows—up-regulation; blue arrows—downregulation. Experimentally validated miRNAs are shown alongside with their target genes in altered expression in CRC

Colorectal cancer is the occurrence of abnormal growth in the colon or rectum. It is the fourth most common cause of cancer-related deaths and one of the most ranked type of cancer worldwide [16]. It is the second and third most common cancer in women and men, respectively, and also accounted for about 10% of the total cancer cases worldwide [16]. The cancer begins with an abnormal growth of the cells lining the colon and rectum. These cells divide uncontrollably and rapidly thereby leading to the formation of a non-cancerous growth or benign tumour known as a polyp. The polyp grows gradually and over a period of 10–20 years (Fig. 1) [17]. An adenomatous polyp or adenoma is the most common type, and about one-third to one-half of all individuals will eventually develop one or more adenomas [18]. Although not all polyps give rise to CRC, but CRC is almost always developed from a polyp and all adenomas have the possibility to be cancerous [18]. The possibility that an adenoma will become cancerous increases as it becomes bigger [19]. Cancer arising from the inner lining of the colorectum is called adenocarcinoma, and accounts for approximately 96% of all CRCs [20]. Series of DNA changes in a polyp’s cell result in its the development into malignant tumor over a period of time (Fig. 1). Initially, these cancer cells are confined to the surface of a polyp, but can grow into the wall of the colon or rectum, which eventually spreads to lymph nodes and other organs, such as the liver or lungs [21].

## CRC pathogenesis and miRNA involvement

**Fig. 2:**
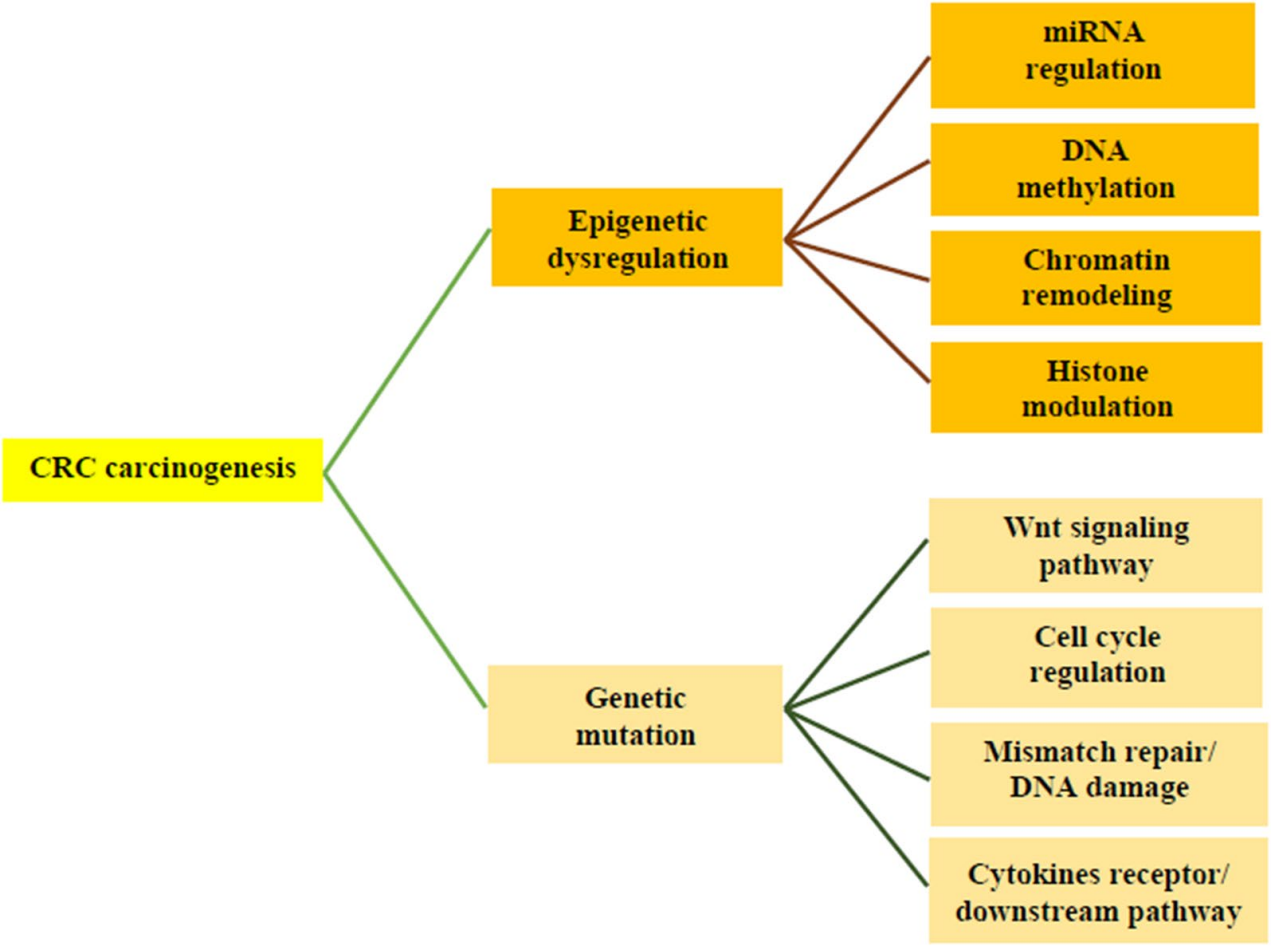
Genetic and epigenetic mechanisms of colorectal carcinogenesis. Mutation of genes involved in the Wnt signaling pathway plays a superior role in colorectal carcinogenesis. Genes that are related to cell cycle progression. DNA repair, and cytokine signaling have also been shown to be pivotal in colorectal carcinogen. DNA hypermethylation of tumor suppressor gene promoter regions has been intensively studied to demonstrate its critical role in gene silencing. Histone modification includes histone methylation and deacetylation, both of which have been shown to be associated with DNA methylation

The molecular mechanism of colorectal carcinogenesis is a multistep process involving genetic, epigenetic and aberrant immunologic pathway as a major contributor of colorectal carcinogenesis [22–24]. miRNAs are often dysregulated in tumors either by genetic or epigenetic factors, and are currently being investigated for their potential as biomarkers in cancer diagnostics (Fig. 2) [25]. This non-coding RNA has been implicated in the mechanism by which gene expression of various cancer-associated genes are controlled and their expression may be altered in the process. A series of studies have highlighted the role of miRNAs in the development of this disease. CRC-related miRNAs have been demonstrated to regulate the genes by various mechanisms, including epigenetic modifications, long non-coding RNA–miRNA, and long non-coding RNA–protein interactions, and by their actions as miRNA precursors. Since miRNAs can be detected in human body fluid and have good specificity and accessibility, they have been suggested to be used as novel potential biomarkers for CRC diagnosis and prognosis as well as in the prediction of the response to therapy [26]. miRNAs have been implicated in a number of events, such as epigenetic, transcriptional, and post-transcriptional regulation [27]. These non-coding RNAs exhibit unique profiles in various human cancers such as colorectal cancer, reflecting disease progression [28]. Studies have previously reported the involvement of miRNAs in cancer initiation and progression but recently, their roles as drivers of tumor suppressor and oncogenic function have been evaluated in several cancer types [29]. Several studies have also shown the association of non-coding RNAs in colorectal carcinogenesis through the stimulation or inhibition of apoptosis, cell proliferation, differentiation, invasion and metastasis [30–35].

## Genetic regulation of colorectal carcinogenesis

Genetic instability has been considered fundamental to the multistep process of tumor growth and metastatic progression for decades [36]. A wide range of genetic mutations is found in most cancer subtypes (Fig. 2). The most common gene mutation in CRC is the APC gene from the normal epithelium followed by the K-ras, DCC and p53 genes leading to sporadic carcinoma [37]. DNA hypermethylation of tumor suppressor gene promoter regions has been intensively studied to demonstrate its critical role in gene silencing (Fig. 2). Histone modification includes histone methylation and deacetylation, both of which have been shown to be associated with DNA methylation [22].

## Epigenetic modifications and miRNA in CRC

Epigenetic alterations have the ability to deregulate the expression of any type of transcript. However, the exact mechanisms of epigenetic regulation of non-coding RNAs are still unclear although, these RNAs are subject to the same epigenetic regulatory mechanisms as protein-coding genes. Several studies have reported the regulatory mechanism of miRNA to clarify the network that underlie the aberrant expression in tumor metastasis. Furthermore, aberrant epigenetic regulation affects abnormal miRNA expression in cancers. miR-21, miR-106, and miR-144 were reportedly upregulated in patients samples with CRC compared with normal individuals [38]. miR 143 and miR-145 were significantly downregulated in colorectal adenoma compared to normal colon sample [39]. These miRNAs were further confirmed to be significantly reduced in colorectal neoplasia and act as tumor suppressor miRNAs in the colorectum [40–44]. Zhang et al. [45] also revealed the induction of apoptosis through BCL-2 inhibition by miR-148a upregulation in CRC while the downregulation was linked to increased tumor size [46]. Attenuated miR-34a and miR-200c expression are associated with metastasis in CRC [47, 48]. Lujambio et al. [49] identified cancer-specific CpG island hypermethylation of the promoter lesion with the transcription of miR-148a, miR-34b/c, and miR-9. miR-34a also have effect on colorectal cancer invasion and metastasis in conjunction with IL-6R, ZNF281, MET, snail family zinc finger 1 and 2 (SNAI1, SNAI2) and β-catenin (CTNNB1) [47, 50–52].

## Overview of miRNA

Micro RNAs are short single stranded non-coding RNAs, consisting of about 19–25 nucleotides. They are responsible for the regulation of translation of genes by binding to the 3′-untranslated region of target mRNAs through sequence-specific manner. These miRNAs reportedly play vital roles in inflammation and carcinogenesis, which can be linked to their oncogenic or tumor suppressive properties [53]. Alterations in miRNA expression are implicated in different human cancers, which include breast cancer, CRC, liver cancer and lung cancer [54]. For gene silencing, cells can use miRNA, which binds and represses messenger RNA (mRNA), thereby turning off genes that are not required in translating genetic information into proteins. This miRNA participates in the regulatory mechanisms of cell’s development through death, and the dysregulation can be implicated in several diseases such as cancer and heart diseases [55].

miRNAs have been recognized as potential biomarkers for early detection, as well as prognostic and therapeutic approach for CRC because of their high level of specificity and selectivity.

## Synthesis of miRNA

As earlier mentioned, miRNA is an important class of post-transcriptional regulators of about 22 nucleotides in length [56], and it carries out its biological functions by binding to the 3′ untranslated regions (UTRs) of its target messenger RNA/s (mRNA/s), thereby repressing its expression [57]. A single miRNA may regulate multiple targets and thus act as a chief controller of gene expression. Human genes (about 30%) can be regulated by miRNAs as suggested by bioinformatic analysis, despite the constitution of 1–3% miRNA of the human genome [58]. Several miRNA-coding genes operate as independent transcription units, which contain their own promoters and regulatory elements. However, about a quarter of miRNA genes are intronic and transcribed alongside their host genes [59].

**Fig. 3:**
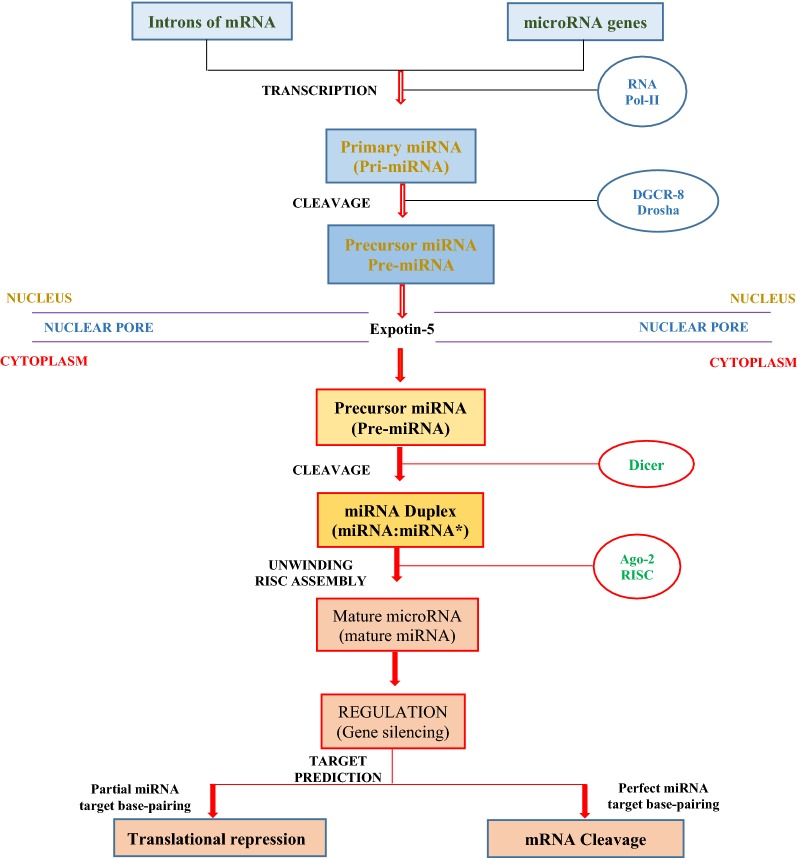
The schematic diagram shows the synthesis and mode of regulation of miRNA from the nucleus to the cytoplasm. The spherical cycles indicate the proteins/enzyme and co-factors responsible for the synthesis of the molecule. Also, the big spherical cycle at the beginning of the reaction indicated that the enzyme is responsible for both mRNA and miRNA synthesis. *DGCR-8* DiGeorge syndrome Critical Region 8 protein, *AGO-2* argonaute protein

Like proteins, genes coding for miRNAs are contained in the nucleus. miRNA can be synthesized from the introns of a functional gene coding for a specific mRNA or from its own gene (Fig. 3). The same enzyme that produces mRNA (RNA polymerase II) transcribes each gene of coded miRNA resulting in a primary miRNA (pri-miRNA), which consists of a 5′ G-cap, at least an approximately 60–70-nucleotide hairpin structure and a 3′ poly (A) tail [60]. Polycistronic pri-mRNA may contain up to seven hairpin structures that produce different mature miRNAs. This pri-mRNA is the final microRNA with regulatory function after several steps. After transcription, the double-stranded stem is recognized by the cofactor DiGeorge syndrome Critical Region 8 protein (DGCR8). An enzyme (Drosha) associates with DGCR8 to form a microprocessing complex capable of cutting the pri-miRNA into a smaller precursor miRNA (pre-miRNA) by the removal of 5′ cap, the 3′ poly (A) tail and sequences flanking the hairpin structure. Precursor-miRNA is then moved from the nucleus through the nuclear pore to the cytoplasm with the aid of Exportin 5, moves where it inactivates mRNA of one or multiple genes [61]. In the cytoplasm, the stem-loop of the pre-miRNA is further cleaved by a large microRNA protein called dicer (an endoribonuclease) to form a short double-stranded microRNA molecule (about 20–25 nucleotides long) consisting of mature miRNA strand and its complementary strand [62].

Consequently, argonaute protein (AGO-2) interacts with dicer to bind the mature miRNA due to the asymmetric thermostability. The miRNA is unwound, and one strand is released (the passenger strand). The guide strand interacts with AGO-2 (responsible for the endonuclease activity that induces mRNA cleavage) and some additional proteins to form the RNA Induced Silencing Complex (RISC). This is guided to the mRNA target to activate one or multiple genes [63]. The mRNA of a target gene is complementary to the sequence of the miRNA that enables base pairing. Complete and partial complementarity between the seed region (nucleotide positions 2–8) of miRNA and its target mRNAs results in mRNA degradation and translational inhibition or repression, respectively [64]. In the case of translational inhibition or repression, the RISC complex prevents the ribosome subunit from binding. The mechanism by which RISC induces translational repression is more complex and may include cap-dependent inhibition of translation initiation, eukaryotic translation initiation factor-6 recruitment to RISC, nascent protein degradation, ribosomal drop-off and prevention of the interaction between poly (A)-binding proteins and eukaryotic translation initiation factor-4G subsequent to mRNA deadenylation [64, 65]. In both cases (mRNA degradation and translational inhibition or repression), the mRNA will not be translated into a protein and the gene is silenced. Since their discovery in the 1900s, major parts of the miRNA’s pathways still remain unclear. However, with their essential role in many biological processes (metabolism, stem cell division, development, apoptosis, cell proliferation, cell cycle control and cell stem differentiation), mRNA offers great potential in medicine and might lead to key treatment of various diseases in the future.

## General functions of miRNA

Several biological functions of miRNA have been reported to be related to various disease mechanisms, regulation of cellular activities and cancer progression [66–69].

Seed region of about 2–8 nucleotides base pair allows miRNAs to bind at different degrees of complementarity. These therefore enables the recognition and binding of a variety of mRNAs which potentially regulate translation and expression of its protein products. Any change in the levels of a specific miRNA expression affects several biological pathways. Partial base pairing inhibits translation without interfering with the integrity of mRNA [70]. The observed discrepancies between mRNA and protein expression levels may be explained by the miRNA action, and the information on miRNA expression and function suggests the regulation of protein expression.

miRNAs are involved in various biological activities including cell differentiation, proliferation, apoptosis, and migration, which are key regulators in various pathogenesis and progression of different diseases, especially cancers [71–73]. miR-15 and miR-16, the first two miRNAs associated with cancer, play a significant role in the regulation of apoptosis by targeting the anti-apoptotic bcl-2 mRNA [74]. Also, the expression of human Ras, regulated by let-7 in cell culture, was also reported as the first miRNA-target interaction with relevance to cancer [75]. Subsequently, numerous publications have reported the role of miRNAs in tumors [76–80].

## miRNA tumor-specific metabolic reprogramming

Cancer cells are shown to experience characteristic changes in their metabolic programs suggesting that metabolic shifts supports tumor cells growth and survival [81]. Report have it that the miRNA expression patterns in human cancers are not the same and that different cancer types have distinct expression patter [82]. This is so because the processing of primary miRNA transcripts to mature RNA is transcribed by RNA polymerase II (Fig. 3). This RNA polymerase II is also responsible for the transcription of mRNAs. Several alterations in miRNA levels have been revealed between colorectal cancer and normal colonic mucosa [83–85]. Gao et al. [86] reported that the c-Myc oncogenic transcription factor, which is known to regulate microRNAs and stimulate cell proliferation, transcriptionally represses miR-23a and miR-23b, resulting in greater expression of their target protein. Interestingly, c-Myc directly binds to the transcription subunit of microRNA (miR)-23a/b and subsequently contributes to the up-regulation of mitochondrial glutaminase 1 via the induction of ASCT2/SLC1A5 transporter. Moreover, the association of c-Myc with miR-17-92 cluster has been shown to inhibit the activity of phosphatase and tensin homologue deleted on chromosome 10 (PTEN) and activates PI3K-Akt-mTOR axis leading to cell survival in early stage adenoma in CRC [87]. The complex crosstalk between miRNA and Myc is considered to be partially responsible for metabolic reprogramming. In addition, metformin induces miR-27b-mediated suppression of ENPP1, which reduces chemoresistance and tumor seeding potential [88].

## Expression of specific miRNAs in cancer

Understanding the deregulation of miRNA expression observed in cancer cells is crucial. Studies have confirmed that when a miRNA is down-regulated in cancer and targets an oncogene, it may act as a tumor suppressor, or may act as an oncogene when up-regulated and targets a tumor suppressor or a gene important for differentiation [89–91].

Carden et al. [92] reported that increased miR-663 expression in breast tumors consistently correlates with increased patient survival, which demonstrates its epigenetic regulation and role in breast tumorigenesis. Also, miR-663a down-regulation was observed in human non-small cell lung cancer progression by targeting AP-1 component JunD in the cancer cells [93]. miR-34a, a chief regulator of tumor suppression, maintains its own expression levels through upstream signaling and activate tumor suppressor pathways, which are regulated by p53 [94]. Wiggins et al. [95] reported that this miRNA inhibits cancer cells lacking endogenous p53.

miRNA has also been implicated in the repression of over 700 transcripts associated with cellular proliferation, survival, and plasticity [96]. High expression of miR-21 predicts poor survival in CRC patient [97–101]. In a contradicting report of Lee et al. [102], the expression of miR-21 in the periphery of primary tumors demonstrated the significance of miRNA as a better prognosis in patients with advanced stage CRC. Molecular validation result of miR-22 expression revealed a significant increase in gastric cancer tissues when compared to adjacent non-cancerous tissues, and that low expression of miR-22 is associated with aggressive gastric cancer phenotype and its poor survival [103]. As suggested in previous studies, miR-22 is associated with several cellular processes, and their deregulation is a hallmark of several human cancers such as ovarian, prostate, colon and liver cancers [104–106]. James et al. [107] also reported the clinical utility of miR-21 and let-7g in prostate cancer. Li et al. [108] investigated the level and role of miR-106a expression in pancreatic cancer and reported that pancreatic cancer cell invasion was dependent on miR-106a regulation [109, 110].

## Diet interaction with microRNAs in colorectal cancer

**Fig. 4:**
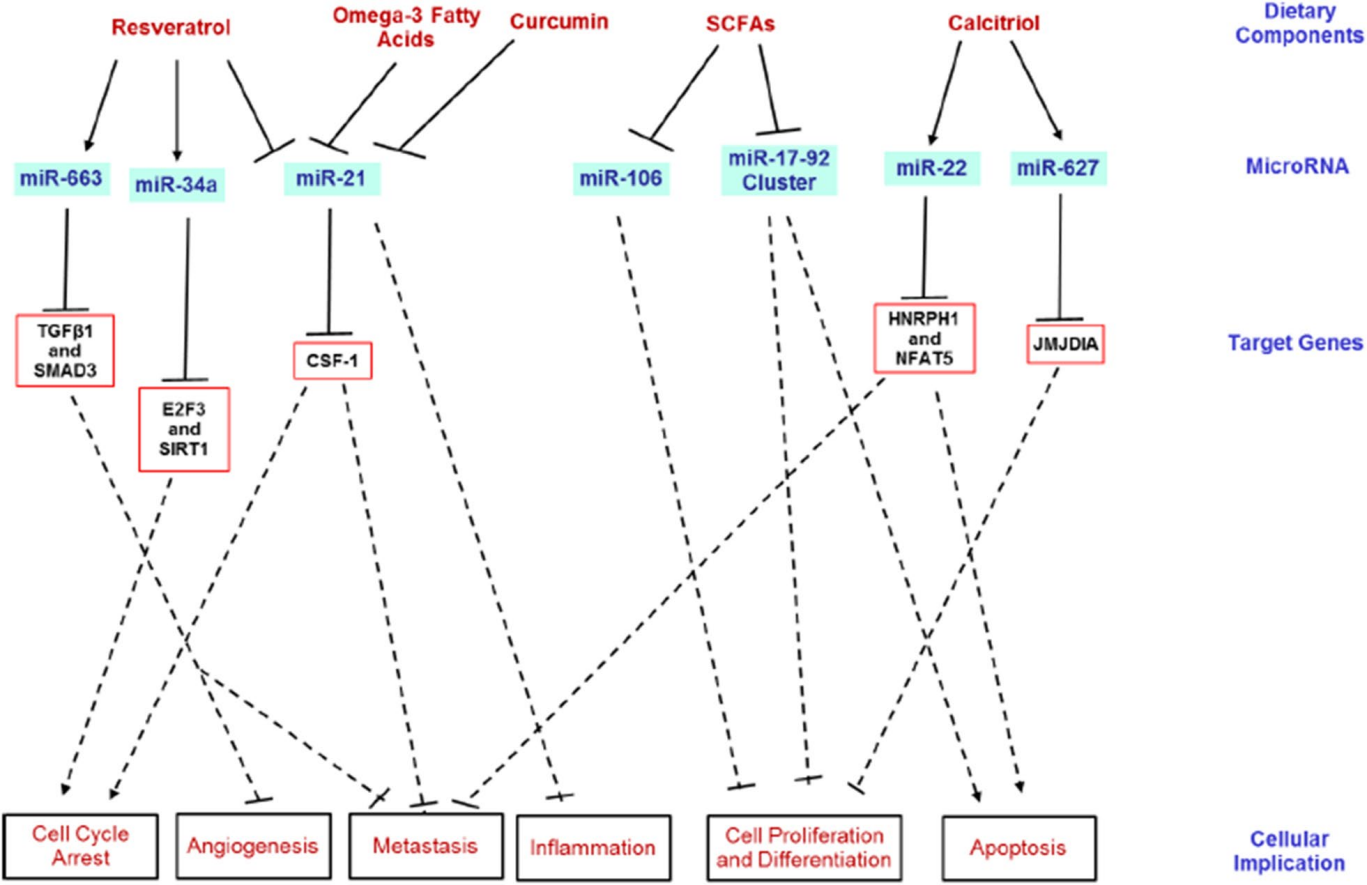
Dietary modulation of microRNAs in colorectal cancer. Several dietary components show chemo-preventive and therapeutic potential in CRC pathogenesis through the modulation of miRNAs in difference signaling pathways. Line arrows indicate up-regulation; blunted lines with flat heads indicate inhibition, while dotted lines indicate multiple steps. *CSF*-*1* Colony stimulating factor 1, *E2F3* E2F transcription factor 3, *HNRPH1* heterogeneous nuclear ribonucleoprotein H1, *JMJDIA* Jumonji domain-containing protein 1A, *NFAT5* nuclear factor of activated T-cells 5, *SIRT1* Sirtuin (silent mating type information regulation 2 homolog) 1, *SMAD3* mother against decapentaplegic homolog 3, *TGFβ1* transforming growth factor beta 1

Research on the discovery of drugs for the treatment of cancer is still ongoing, with several shortcomings due to the complex genetic and epigenetic events involved in its pathogenesis. However, strong evidence continues to show that certain dietary components possess cancer-protective capabilities, including therapeutic and chemopreventive properties. These dietary factors may play a role in several stages of carcinogenesis, such as cell-cycle modulation, inflammation, apoptosis, DNA repair and angiogenesis [111]. miRNAs are intrinsically involved in similar stages of carcinogenesis, which widens the understanding between miRNAs and certain dietary components (Fig. 4). Certain dietary components of plant origin may be less bio-available and thus, escape digestion into the large intestine. Therefore, these bioactive components may then play a role in modulating CRC.

### Resveratrol

Resveratrol, polyphenols found largely in the skin of grapes, raspberries, mulberries, and blueberries, are generally considered to have several health benefits due to its antioxidative properties. Few studies have shown the potential role of resveratrol against colon cancer. Treatment of SW480 colon cancer cells with 50 µM of resveratrol for 14 h prevented the expression of several oncogenic miRNAs, such as miR-21 which is induced in chronic inflammation [112]. Furthermore, the expression of a tumor-suppressor miRNA, miR-663, was significantly higher in cancer cells when its expression was compared to untreated cells. The use of resveratrol in the treatment of colon cancer cells has led to a reduction in TGFβ1 and its downstream effector SMAD3, this could be explained by the target of miR-663 on TGFβ1 transcripts [112]. This finding on miR-663 is of significance since activation of the TGFβ1 pathway increases angiogenesis and metastasis in later stages of cancer [112, 113]. In addition, resveratrol was also shown to inhibit the up-regulation of miR-122, an oncogenic and inflammation-induced miRNA, which is dependent manner on miR-663 [114]. Another study showed that resveratrol inhibited the growth of human colon cancer cells by up-regulating miR-34a, which in turn down-regulated the E2F3 and Sirt1 genes [115]. Therefore, resveratrol possesses anti-inflammation and anti-cancer capabilities, which might be linked to its antioxidative properties.

### Fatty acids

Short-chain fatty acids (SCFAs) and omega-3 fatty acids have been shown to have cancer-protective properties. Short-chain fatty acids are important end products of the gut microbial fermentation of non-digestible carbohydrates in the diet. Butyrate most importantly is vital for the proliferation and sustenance of colonic epithelial cells. As such, several studies have reported the involvement of microbial-derived butyrate from dietary fiber fermentation as a histone deacetylase (HDAC) inhibitor and thus plays a protective role against colorectal tumorigenesis [116–118]. However, the chemoprotective effect of butyrate on specific miRNAs remains to be fully elucidated. In human colon carcinoma cells, microarray analysis revealed that treatment with 2 mM butyrate changed the levels of various aberrantly expressed miRNAs [119]. Notably, butyrate treatment decreased the expression of miR-106a and miR-106b, which was accompanied by a reduction in cell proliferation [119]. Furthermore, in other human colon cancer models, treatment with 1–25 mM butyrate attenuated the expression of an oncogenic miR-17-92 cluster of miRNAs, while inhibiting cell differentiation and promoting apoptosis [120, 121].

Omega-3 fatty acids may have protective effect against inflammatory diseases, including cancer [122, 123]. Specifically, it was reported that fish oil prevented the down-regulation of several miRNAs in the colon of rats 34-weeks post-injection with azoxymethane. Such miRNAs include miR-15b, miR-107, let-7d, miR-191, and miR-324-5p. This effect corresponds to a significant reduction in colon tumorigenesis [124]. Similarly, the expression of miR-21 was significantly diminished in breast cancer cell lines treated with fish oil, thus repressing CSF-1 levels which have a significant role in breast tumorigenesis and metastasis [125]. Put together, these findings strongly suggest the chemo-preventive potential of SCFAs and omega-3 fatty acids (that could be obtained through the diet).

### Curcumin

Curcumin, a phytochemical found in turmeric, has been widely studied for its several health benefits, including antioxidant, anti-inflammatory and anti-cancer properties. Studies have also reported miRNA modulation in various cancer models. Recently, curcumin was reported to reduce the expression of miR-21, which is over-expressed in many tumors leading to cancer progression and metastasis [126]. Treatment of human colon carcinoma cells (HCT-116) with curcumin reduced miR-21 activity in a dose-dependent manner, thereby leading to cell cycle arrest at the G_2_/M phase, thus reducing cell proliferation and tumor growth [126]. A similar effect of curcumin on miR-21 was also reported in a pancreatic cancer cell model [127]. Other studies have also reported the beneficial effect of curcumin in miRNA modulation in various cancer models, including pancreatic cancer [127–129] and lung cancer [130].

### Vitamin D

Vitamins A, D, and E have been reported to play an anti-cancer role involving the modulation of miRNAs [131, 132], amongst which vitamin D have an active chemo-preventive role in CRC development. Early epidemiological evidence suggested an inverse relationship between vitamin D levels and CRC [133]. Further studies in human colon cancer cells revealed that treatment with 10 µM calcitriol (an active form of vitamin D) induced miR-22 expression which further inhibits cell proliferation and migration. These effects were time- and dose-dependent, and also dependent on the activation of vitamin D receptor [134]. Moreover, up-regulation of miR-22 by vitamin D in the colon cancer cells is necessary for the repression of several vitamin D target genes, such as HNRPH1 and NFAT5, which mediate apoptosis inhibition and cancer invasion, respectively [134]. In addition, the expression of miR-627 was up-regulated following incubation of human CRC cells (HT-29) with calcitriol, which down-regulates JMJD1A (a gene involved in histone methylation), and prevent cell proliferation and differentiation [135]. Thus, current knowledge posits that vitamin D has cancer-suppressive potentials, which may be mediated via microRNA activation.

### Selenium

Selenium is an essential trace mineral with an antioxidant activity, which was shown to be beneficial in promoting cardiac health and preventing cancer development [136]. Although its role in cancer prevention has been widely reported [136], little is known about its effect on miRNA activity in cancer models. Of note, incubation of human prostate cancer cells (LNCaP) with sodium selenite (2.5 µM) up-regulated members of the miR-34 family, resulting in a selenium-induced expression and activation of the tumor-suppressor p53, and its downstream targets [137]. Other metabolites of selenium, including methylselenocysteine and selenomethionine, have been found to possess HDAC-inhibiting activity in human colon cancer cells [138], but the knowledge of possible miRNAs involved is still vague.

### Soy isoflavones

Diadzein, genistein, and glycitein are soy isoflavones that were reported to have anti-tumor properties via the modulation of the estrogen receptor [131]. Their chemo-preventive and anti-metastasis potential via the modulation of miRNAs was reported in pancreatic cancer [139], prostate cancer [140], and ovarian cancer [141] models. It is interesting to investigate the potential role of the soy isoflavones in colon cancer, since these isoflavones act via the modulation of estrogen receptor. It was suggested that an up-regulation of the estrogen receptor beta (ERβ) signaling in SW480 colon cancer cells showed antiproliferative effects by silencing the effect of oncogenic miRNAs [142].

### Ellagitannin

Ellagitannins are hydrolyzable polymeric polyphenols found in many fruits and nuts. Initial characterization of ellagitannins showed their potent antioxidant, anti-inflammatory, anti-proliferation and pro-apoptotic capabilities [143]. More recently, ellagitannin was shown to possess anti-neoplastic properties in a human liver cancer cell line HepG2, while modulating the expression of 25 miRNAs [144]. However, the specific mechanisms of the ellagitannin-miRNA interplay in cancer is still unknown.

### Caloric restriction

Caloric restriction (CR) generally refers to a ≤ 60% dietary energy deficit without malnutrition [145]. The beneficial effects of CR have been reported in various conditions, including aging and cancer. CR has long been known to play a vital role in colon cancer prevention, but specific mechanisms and miRNAs involved still requires further evaluation [146, 147]. The anti-cancer effects of CR may be due to its influence on cellular senescence [148]. The Hayflick limit, which described cellular senescence as a stable cell cycle arrest regardless of growth conditions, was thought to protect against the heightened proliferation of cancer cells [149, 150]. In paradox, senescent cells may also contribute to tumorigenesis in various tissues, through the production of an array of cytokines, chemokines, proteases and growth factors, collectively referred to as the senescence-associated secretory phenotype (SASP) [151, 152]. Unsurprisingly, NFκβ is known to play a role in regulating various inflammatory pathways involved in producing the senescence secretome, that drives the chronic low-grade inflammation capable of driving tumor initiation and progression [153, 154].

Some of the consequences of overnutrition-induced obesity are hyperinsulinemia and hyperleptinemia, resulting in insulin and leptin resistance respectively. These may serve as growth factors leading to the activation of NFκβ, thus leading to chronic inflammation characteristic of many tumors [154–156]. On the other hand, CR may impact the obesity-cancer pathway, by reducing serum insulin, leptin, and associated inflammation by limiting NFκβ—related gene expression [157, 158]. Specifically, injection of mice on a 30% CR diet with MC38 colon tumor cells, led to a reduction in tumor size, serum growth factors and a downregulation of inflammatory genes induced by NFκβ [157]. Similarly, 5-week feeding of a 30% CR diet in mice showed inhibitory effects on pancreatic tumor growth, IGF-1 and NFκβ-related inflammatory gene expression [158]. Still, possible miRNAs involved in the anti-tumor effects of CR in relation to the NFκβ-SASP pathway are still largely unclear. Few breast cancer models have shown that CR may impact miRNAs, by showing inhibitory effects on miR17/20a and miR200a, leading to a reduction in extracellular matrix proteins, tumor progression and metastasis [159, 160]. Put together, it may be hypothesized that CR possesses anti-cancer effects by decreasing chronic inflammation through the limitation of NFκβ activity in senescent cells. However, this concept, potential mechanisms, and miRNAs involved are interesting subjects for future studies. Understanding this effect of CR may be important in preventing colorectal cancer and other cancers in our obese and older adult populations where low-grade inflammation and cellular senescence are more observed, respectively.

## Conclusion

It is now a known fact that CRC is a major depravity that affects the world based on lifestyle changes and sometimes based on age or hereditary factors. Regular screening for CRC is essential and should be done to detect tumor early before it metastasizes. Several screening and treatment methods have been employed for CRC, which have been of help to date but present several limitations. Recently, the involvement of 18–22 nucleotide to the foreknown miRNA, and its relation to dietary factors and tumorigenesis. This microRNA can be differentially and commonly expressed depending on its stage and location of the tumor. The ability of microRNA to differentiate between CRC patients and healthy patients in a non-invasive approach for CRC detection makes it a good diagnostic biomarker. Currently, little is known on the impact of diet on miRNAs in CRC, as most studies were only centered on in vitro models. Studies providing information on the use of miRNA-specific knockout should be considered in various in vivo models. Apart from the few described in this review, other dietary components such as folate and methyl-deficient diets, indoles and isothiocyanates (from cruciferous vegetables) and tea catechins have been widely shown to possess chemo-preventive properties but their effect via the modulation of microRNAs in the colon and rectal cancer is still unclear. Collectively, bioactive components from the diet modulate several miRNAs which are involved in cancer development and growth via several mechanisms. Due to their potent chemo-preventive properties, it is therefore pertinent for public health specialists and health organizations to consider incorporating these dietary components into the nutrition sensitization program to prevent or reduce the menace of CRC and other malignancies.

## Abbreviations

CRC: colorectal cancer
miRNAs: microRNAs
RISC: RNA-induced silencing complex
UTR: the 3′untranslated region
DGCR-8: DiGeorge syndrome Critical Region 8 protein
AGO-2: argonaute protein
ERβ: estrogen receptor beta
SCFAs: short-chain fatty acids
CASP3: cysteine-aspartic acid protease 3
APC: adenomatous polyposis coli
MMPs: matrix metalloproteinases
DCC: deleted in colorectal carcinoma
EGFR: epidermal growth factor receptor
ICAM: intercellular adhesive molecules
PDCD4: programmed cell death 4
PTEN: phosphatase and tensin homolog
CDK4,6: cyclin-dependent kinase 4,6
ECM: extracellular matrix
EMT: epithelial-to-mesenchymal transition
RECK: reversion-inducing cysteine-rich protein with kazal motifs
TIMP3: tissue inhibitor of metalloproteinase 3
uPAR: plasminogen activator, urokinase receptor
TGFβRI/II: transforming growth factor βreceptor I/II
ZEB1/2: zinc-finger E-box binding homeobox-1
CTGF: connective tissue growth factor
TSP1: thrombospondin-1
